# Inequalities by education and marital status in the co-occurrence of cardiovascular risk factors in Finland persisted between 1997–2017

**DOI:** 10.1038/s41598-020-65959-1

**Published:** 2020-06-04

**Authors:** Laura Paalanen, Tommi Härkänen, Jukka Kontto, Hanna Tolonen

**Affiliations:** Department of Public Health Solutions, Finnish Institute for Health and Welfare (THL), P.O. Box 30, FI-00271 Helsinki, Finland

**Keywords:** Biomarkers, Risk factors

## Abstract

Understanding on sociodemographic variation of the co-occurrence of cardiovascular disease risk factors is crucial for planning future prevention strategies. We aimed at examining (1) the co-occurrence of smoking, obesity, hypertension and elevated serum cholesterol by education and marital status, and (2) its trends in different sociodemographic groups in Finland. We used data from cross-sectional health examination surveys among the general population (25–64 years): for 1997–2012 the National FINRISK Study and for 2017 the FinHealth 2017 Survey (n = 25036). A risk factor accumulation score with categories (1) zero, (2) one, (3) two, and (4) three or four elevated risk factors was the outcome in multinomial logistic regression. The risk factor score was more favourable among women, among high education groups, and slightly among participants living with a spouse. Among men, the lowest risk factor score class became more prevalent especially in the intermediate education group, which approached the highest education group over time. Our results indicate an overall transition towards a more favourable risk factor distribution. However, risk factor accumulation among the least educated remained emphasizing the need to develop and implement more targeted prevention interventions and public health policies to decrease the risk factor burden particularly in this group.

## Introduction

Despite a decrease in mortality in diseases of the circulatory system in Finland over the past decades, every fifth death for men and every sixth death for women was still due to ischaemic heart disease (IHD) in 2017^1^. Furthermore, the decrease in IHD mortality has been more rapid in higher socioeconomic groups, and thereby, the socioeconomic inequality has increased^2^. At the same time, high blood pressure and smoking were the leading risk factors of death globally^3^.

The co-occurrence of major chronic disease risk factors and its association with a broad range of health outcomes such as mortality, life expectancy, onset of cardiovascular disease (CVD) and health-related quality of life has been reported in literature^4–8^. Some studies have concentrated on lifestyle risk factors, such as smoking, physical activity, diet and alcohol use^9^. In other studies, biological risk factors, such as serum cholesterol, blood pressure and diabetes status have been included in addition to lifestyle risk factors^4,6^. As risk factors are often associated, the patterns of clustering of several risk factors have also been analyzed. Systematic reviews have found that alcohol misuse and smoking is the most commonly identified risk behavior combination among adults^9,10^. The understanding on how risk factors link to each other may be used in, for example, planning interventions targeting simultaneously on several commonly co-occurring risk factors in specific population groups. As many chronic diseases have common risk factors, such interventions would have a favourable effect on many diseases. Thereby, many lifestyle interventions have also been calculated to be cost-effective considering the costs of the intervention in relation to treatment of outcome disease^11,12^.

The understanding on sociodemographic variation of the co-occurrence of risk factors, either biological risk factors or unhealthy lifestyles, is crucial for planning public health policy and future prevention strategies. Male sex and lower socioeconomic status, such as lower level of education, occupation or income, have been shown to have the most consistent relationship with co-occurrence or clusters of health related risk behaviours^9,10^ revealing that the risk behaviours tend to accumulate in lower socioeconomic groups.

Although vast literature on the co-occurrence of risk factors exists, the temporal changes in the co-occurrence or, more specifically, in the sociodemographic variation or the tendency of the risk factors to accumulate in lower socioeconomic groups have not been extensively studied. In Finland, the trends in the variation in unhealthy behaviour among Finnish men from 1982 to 1990 have been examined, and unhealthy behavior was especially common among divorced men with a low educational level^13^. However, the degree of accumulation did not change during this short period of roughly ten years.

We aimed at examining the co-occurrence of major CVD risk factors, namely smoking, obesity, hypertension, and elevated serum total cholesterol, by education and marital status using data from large population-based surveys among adults covering years 1997–2017 from Finland. We also aimed to examine temporal changes in the sociodemographic differences in the co-occurrence of these risk factors.

## Methods

### Study population

This study is a part of a research project ‘Projections of the burden of disease and disability in Finland’^14^. The data comprise a series of cross-sectional health examination surveys carried out every five years in Finland covering past 20 years. For the time period 1997–2012, data from the National FINRISK Study were used^15^, in which the sample sizes were in total 35835 (Table 1). Five study areas from the National FINRISK Study are included in the analyses: North Karelia, Northern Savo, cities of Turku and Loimaa with selected surrounding municipalities, cities of Helsinki and Vantaa, and regions of Northern Ostrobothnia and Kainuu (also known as the Oulu region).

**Table 1:** Data characteristics.

	National FINRISK surveys 1997–2012				FinHealth 2017
	1997	2002	2007	2012	2017
***Men***					
Sample	5000	4972	3983	3960	2346
Participants (n)	3395	3247	2412	2276	1411
Participation rate (%)	68	65 (59^a^)	61 (56^a^)	57 (52^a^)	60 (51^a^)
n, final^b^	3344	2915	2192	2023	1159
***Women***					
Sample	5000	4980	3979	3961	2181
Participants (n)	3763	3763	2821	2660	1524
Participation rate (%)	75	76 (70^a^)	71 (66^a^)	67 (60^a^)	70 (61^a^)
n, final^b^	3704	3460	2582	2350	1307
Education (%)					
*Men*					
Low	31.8	34.3	30.6	33.6	31.1
Intermediate	33.3	31.8	34.5	33.1	33.2
High	34.9	33.9	34.9	33.3	35.7
*Women*					
Low	32.6	33.6	33.2	32.1	30.2
Intermediate	35.6	31.5	32.9	33.1	32.4
High	31.8	34.9	33.9	34.8	37.4
Marital status^c^ (%)					
*Men*					
Married	75.1	75.7	73.8	74.8	74.2
Single	24.9	24.3	26.2	25.2	25.8
*Women*					
Married	73.4	73.9	71.9	71.5	73.0
Single	26.6	26.1	28.1	28.5	27.0
	mean (SD)	mean (SD)	mean (SD)	mean (SD)	mean (SD)
Age, years					
*Men*	45.6 (11.2)	46.1 (11.3)	46.1 (11.2)	46.1 (11.4)	47.1 (11.5)
*Women*	45.0 (11.3)	44.9 (11.5)	45.2 (11.3)	45.5 (11.4)	46.8 (11.5)
BMI, kg/m^2^					
*Men*	26.9 (4.0)	27.2 (4.1)	27.2 (4.2)	27.3 (4.4)	27.6 (4.7)
*Women*	26.1 (5.0)	26.3 (5.1)	26.4 (5.4)	26.3 (5.4)	26.7 (5.4)
Serum total cholesterol, mmol/l					
*Men*	5.6 (1.1)	5.5 (1.1)	5.3 (1.0)	5.3 (1.1)	5.2 (1.1)
*Women*	5.5 (1.1)	5.3 (1.0)	5.2 (1.0)	5.3 (1.0)	5.2 (1.0)
Systolic BP, mmHg					
*Men*	137.0 (17.9)	136.1 (17.7)	136.0 (17.5)	134.0 (16.1)	132.3 (15.8)
*Women*	130.2 (18.8)	130.1 (19.6)	128.8 (18.6)	127.5 (17.1)	125.9 (17.2)
Diastolic BP, mmHg					
*Men*	84.5 (11.4)	81.4 (11.5)	82.0 (11.6)	84.2 (11.0)	82.7 (11.0)
*Women*	79.9 (10.8)	76.5 (11.0)	76.4 (10.7)	78.6 (10.6)	78.3 (10.7)
Current smoking (%)					
*Men*	33.4	35.7	30.8	28.5	24.4
*Women*	21.6	23.6	21.3	20.8	17.8

The sample sizes, participation and crude means and standard deviations (SD) for age, body mass index (BMI), serum total cholesterol and systolic and diastolic blood pressure, and the percentage of current smoking. The age range of included participants was 25–64 years.

BMI = body mass index, BP = blood pressure.

^a^Participation rate for those who returned the questionnaire and went through the health examination.

^b^Number of participants included in the current analyses, i.e. participants for whom data on all risk factor profile variables (current smoking, body mass index, systolic and diastolic BP and serum total cholesterol) were available.

^c^Marital status classes: married = living with a spouse/partner (i.e. married, cohabiting), and single = living without a spouse (i.e. single, separated or divorced, widowed).

In 2017, the sampling was modified somewhat, and the name of the study was changed to the FinHealth 2017 Survey^16,17^. 10000 adults aged 18 years or older and living in Finland were invited to participate by two-stage cluster sampling from the population register. The sample represented the continental Finland. However, to ensure comparability with the FINRISK surveys, we included only the same five study areas for FinHealth 2017 data as for FINRISK surveys 1997–2012.

The age range of subjects of the included surveys has varied across study years. In this study, participants aged 25–64 years were included in the analyses. Furthermore, only participants who attended the health examination component of the surveys with physical measurements and collection of blood samples were included in this study. In total, 25036 participants were included in the analyses (Table 1).

All data have been collected by the Finnish Institute for Health and Welfare (THL), Finland (formerly the National Public Health Institute, KTL). The prevailing legislation and regulations have been followed for all surveys. Survey in 1997 was approved by the Ethical Issues’ Committee of the National Public Health Institute (KTL) (No. 38/96), survey in 2002 by the Ethical Committee for Research in Epidemiology and Public Health at the Hospital District of Helsinki and Uusimaa (HUS) (No. 558/E3/2001) and surveys in 2007, 2012 and 2017 by the Coordinating Ethics Committee for the Helsinki and Uusimaa Hospital District (299/EO/06, 162/13/03/00/2011 and 37/13/02/00/2016, respectively). A written informed consent was obtained from all participants. All survey methods were performed in accordance with the relevant guidelines and regulations.

### Measures

Four major modifiable risk factors for chronic diseases were chosen: current smoking, obesity, hypertension, and elevated serum total cholesterol (Table 1). Smoking was assessed by structured questions in a self-administered questionnaire. We classified the participants into smokers and non-smokers. Smokers were defined using information from a series of smoking related questions. Smokers were persons who reported that they had smoked regularly (almost every day) at least for one year and had not stopped smoking in the past six months. Obesity was defined as body mass index (BMI) of ≥30 kg/m^2^. BMI was calculated on the basis of height and weight, measured during the health examination part of each study. Blood pressure was measured by mercury sphygmomanometers in all surveys according to the same standardized protocol. Hypertension was defined as systolic blood pressure ≥140 mmHg and/or diastolic blood pressure ≥90 mmHg and/or the use of antihypertensive medication. Total cholesterol was determined from blood samples drawn after a minimum of 4-h fast. Different methods have been applied in the determination of serum total cholesterol over the study years. The laboratory in the Finnish Institute for Health and Welfare (THL) has taken part in both national and international quality assurance systems first with the WHO centre in Prague and the last three surveys with the Center for Disease Control and Prevention in Atlanta. Based on the quality diagnostics, the systematic errors for cholesterol values were corrected with respective coefficients for each survey year that have been reported previously^18^. Elevated total cholesterol was defined as serum total cholesterol ≥5.0 mmol/l and/or use of cholesterol-lowering medication. The information on antihypertensive and cholesterol-lowering medication was derived from a self-administered survey questionnaire. Those subjects who reported that they had used antihypertensive medication during the past week, were classified as using medication. For cholesterol-lowering medication, the question was somewhat different, and those who reported “current” use of cholesterol-lowering medication, were included.

The number of these risk factors was used to categorize the participants into four risk factor accumulation categories: 1) no risk factors, 2) one risk factor, 3) two risk factors, and 4) three or four risk factors. Henceforth, this four-category outcome variable will be referred to as the risk factor accumulation score.

### Marital status and education

Gender, marital status and education were selected as sociodemographic indicators. Marital status was dichotomized to 1) living with a spouse/partner (married, cohabiting), and 2) living without a spouse (single, separated or divorced, widowed). Education was based on the self-reported total number of years of formal education. The educational attainment of the participants was classified as low, intermediate or high by dividing years of formal education into tertiles within each birth cohort, separately for men and women.

### Statistical analyses

All analyses were performed separately for men and women. Unweighted means and prevalences of sociodemographic indicators and risk factors are shown in Table 1. All other analyses have been performed using poststratification weights calibrated using age, sex, area and study year.

The age-adjusted prevalences for all possible risk factor combinations (16 combinations) of the four binary factors were analyzed using a multinomial logistic regression model (MLR) (Tables 2 and 3). The covariates were age and study year as categorical variables. If the Wald test result was found significant, then the changes in prevalences of the single risk factor combinations were further examined using 16 binary logistic regression models, in which the outcome was defined as having one of the 16 combinations or not.

**Table 2:** Prevalence of risk factor combinations (%) among 25–64-year-old men in FINRISK (FR) 1997–2012 and FinHealth (FH) 2017 surveys (n = 11,633).

Number of risk factors	Current smoking	Obesity^a^	Elevated cholesterol^b^	Hypertension^c^		FR97	FR02	FR07	FR12	FH2017	p
4	+	+	+	+		2.8	3.8	2.6	3.9	3.9	0.06
					Total	2.8	3.8	2.6	3.9	3.9	
3	+	+	+	−		1.5	1.7	1.9	1.5	0.7	0.45
	+	+	−	+		0.6	0.7	0.7	0.7	1.0	0.90
	+	−	+	+		9.4	8.5	7.8	5.7	4.0	<0.001
	−	+	+	+		7.3	7.2	7.2	7.9	7.5	0.91
					Total	18.8	18.1	17.6	15.8	13.2	
2	+	+	−	−		0.3	0.8	0.7	0.6	1.1	0.32
	+	−	+	−		10.5	12.0	8.6	8.3	4.7	<0.001
	+	−	−	+		2.7	2.0	2.3	2.5	1.8	0.34
	−	+	+	−		2.3	3.2	2.5	2.1	4.6	<0.01
	−	+	−	+		1.8	1.9	2.6	2.2	2.3	0.30
	−	−	+	+		19.0	13.4	16.5	17.4	14.7	<0.001
					Total	36.6	33.3	33.2	33.1	29.2	
1	+	−	−	−		5.4	7.3	7.3	5.6	6.3	0.06
	−	+	−	−		0.7	1.6	0.5	1.6	2.4	<0.001
	−	−	+	−		17.1	18.5	18.1	18.7	22.2	0.06
	−	−	−	+		5.8	5.0	6.7	5.2	5.2	0.21
					Total	29	32.4	32.6	31.1	36.1	
0	−	−	−	−		12.6	12.5	14.0	16.1	17.9	0.001
					Total	12.6	12.5	14.0	16.1	17.9	

^a^BMI ≥ 30 kg/m^2^.

^b^Serum total cholesterol ≥5.0 mmol/l or use of cholesterol-lowering medication.

^c^Systolic blood pressure ≥140 mmHg or diastolic blood pressure ≥90 mmHg or the use of antihypertensive medication.

**Table 3:** Prevalence of risk factor combinations (%) among 25–64-year-old women in FINRISK (FR) 1997–2012 and FinHealth (FH) 2017 surveys (n = 13,403).

Number of risk factors	Current smoking	Obesity^a^	Elevated cholesterol^b^	Hypertension^c^		FR97	FR02	FR07	FR12	FH2017	p
4	+	+	+	+		1.4	1.2	1.6	2.0	1.3	0.44
					Total	1.4	1.2	1.6	2.0	1.3	
3	+	+	+	−		1.4	1.7	1.4	1.0	2.3	0.11
	+	+	−	+		0.1	0.4	0.9	0.4	0.1	0.11
	+	−	+	+		3.7	2.7	2.4	2.9	2.6	0.20
	−	+	+	+		8.0	6.6	6.7	7.8	6.7	0.13
					Total	13.2	11.4	11.4	12.1	11.7	
2	+	+	−	−		0.7	0.9	0.9	0.5	1.1	0.46
	+	−	+	−		8.6	7.6	6.5	6.3	4.8	0.001
	+	−	−	+		1.0	1.3	1.2	0.6	1.0	0.31
	−	+	+	−		3.3	3.6	3.6	4.5	5.8	0.01
	−	+	−	+		1.5	1.3	1.9	1.1	1.6	0.26
	−	−	+	+		13.6	12.0	11.0	12.1	9.4	<0.01
					Total	28.7	26.7	25.1	25.1	23.7	
1	+	−	−	−		7.0	8.6	8.1	7.0	6.0	0.12
	−	+	−	−		1.6	2.0	2.2	2.5	3.2	0.08
	−	−	+	−		25.3	23.7	21.9	24.9	25.5	0.05
	−	−	−	+		3.5	4.5	5.3	3.7	2.8	<0.01
					Total	37.4	38.8	37.5	38.1	37.5	
0	−	−	−	−		19.3	21.8	24.6	22.6	25.8	<0.001
					Total	19.3	21.8	24.6	22.6	25.8	

^a^BMI ≥ 30 kg/m^2^.

^b^Serum total cholesterol ≥5.0 mmol/l or use of cholesterol-lowering medication.

^c^Systolic blood pressure ≥140 mmHg or diastolic blood pressure ≥90 mmHg or the use of antihypertensive medication.

**Figure 1:**
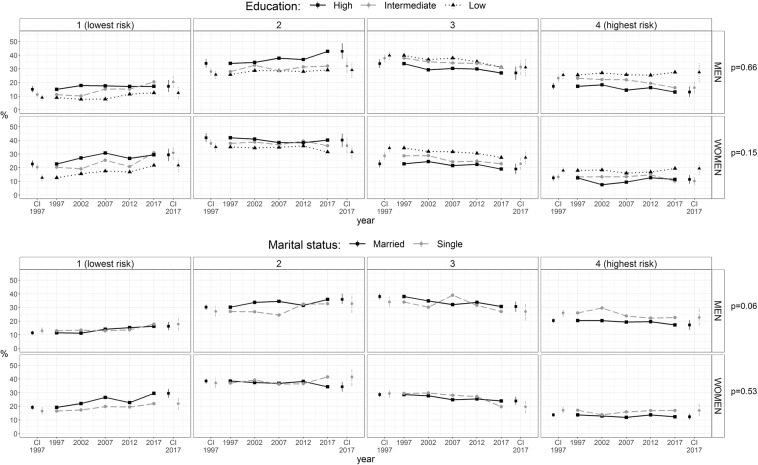
Risk factor accumulation score by education and marital status and p values for interaction between education and study year or education and marital status. The risk factor accumulation score was formed from the following four risk factors: current smoking, obesity, elevated total cholesterol and hypertension. The classes were as follows: (1) no risk factors, (2) one risk factor, (3) two risk factors, and 4) three or four risk factors. The 95% confidence intervals (CI) are presented for years 1997 and 2017. The number of men and women in the analyses by education were 11540 and 13277, respectively, and in the analyses by marital status 11616 and 13380, respectively.

Further analyses utilized the four-category risk factor accumulation score. To examine sociodemographic differences, MLR was used with the risk factor accumulation score as the outcome. 95% confidence intervals of the estimates were defined as the 2.5% and 97.5% percentile points of the 1000 estimates from the bootstrap samples. More specifically, MLR was applied to estimate predicted probabilities for each education tertile or marital status class within each risk factor accumulation score category adjusted for age and study area (Fig. 1, Tables 4 and 5). Wald test was used to test the significance of the interaction between the sociodemographic variables (education and marital status) and study year in predicting the risk factor accumulation score.

**Table 4:** Risk factor accumulation score^a^ by education in FINRISK (FR) 1997–2012 and FinHealth (FH) 2017 surveys. The age range of included participants was 25–64 years.

Risk factor score^a^	Education level	FR97	FR02	FR07	FR12	FH2017	p for interaction^b^
**Men** (n = 11540)							0.66
1 *lowest risk*	Low	8.9 (7.0–11.1)	7.7 (5.7–9.5)	7.8 (5.5–10.5)	11.4 (8.4–14.3)	12.4 (8.2–16.7)	
	Intermediate	11.1 (8.9–13.5)	10.1 (7.8–12.6)	15.1 (12.0–18.2)	15.1 (12.1–18.7)	20.4 (16.0–24.8)	
	High	15.0 (12.8–17.3)	17.8 (15.3–20.6)	17.5 (14.6–20.6)	17.1 (14.1–20.5)	17.2 (13.1–21.8)	
2	Low	25.8 (22.7–28.7)	28.7 (25.5–32.0)	28.6 (24.6–32.9)	28.0 (24.4–31.8)	29.1 (23.5–35.4)	
	Intermediate	28.0 (24.9–31.1)	32.6 (29.1–36.4)	28.6 (24.8–32.5)	31.4 (27.1–35.7)	32.1 (27.0–38.0)	
	High	34.0 (30.9–37.1)	34.7 (31.4–37.8)	37.8 (33.9–41.7)	36.8 (32.1–41.3)	42.8 (37.3–48.7)	
3	Low	39.8 (36.7–43.3)	36.7 (33.2–40.1)	37.9 (33.5–42.1)	35.3 (31.4–39.7)	31.1 (24.8–37.4)	
	Intermediate	37.8 (34.7–40.9)	35.2 (31.7–38.9)	34.3 (30.5–38.2)	34.0 (29.8–38.1)	31.3 (25.7–36.3)	
	High	33.8 (30.7–36.9)	29.3 (26.0–32.8)	30.3 (26.6–34.1)	29.9 (25.6–34.2)	27.0 (21.9–31.7)	
4 *highest risk*	Low	25.5 (22.7–28.5)	27.0 (23.7–30.2)	25.7 (21.9–29.7)	25.3 (21.6–28.7)	27.5 (20.3–34.3)	
	Intermediate	23.1 (20.4–25.7)	22.1 (19.2–24.8)	22.0 (18.8–25.0)	19.4 (16.1–22.6)	16.1 (12.3–20.3)	
	High	17.2 (14.9–19.6)	18.3 (15.5–20.7)	14.4 (11.6–17.3)	16.2 (13.2–19.3)	13.0 (9.5–17.2)	
**Women** (n = 13277)							0.15
1 *lowest risk*	Low	12.6 (10.5–15.0)	15.5 (13.1–18.1)	17.5 (14.5–20.5)	16.8 (13.7–20.0)	21.7 (17.2–26.6)	
	Intermediate	20.4 (18.2–22.6)	19.1 (16.6–21.7)	25.5 (22.2–28.8)	20.8 (17.2–24.3)	30.9 (25.9–36.0)	
	High	22.7 (20.2–25.3)	27.1 (24.3–29.8)	30.7 (27.5–34.0)	26.8 (23.4–30.1)	29.4 (24.9–33.8)	
2	Low	35.1 (31.9–38.1)	34.4 (31.4–37.6)	34.9 (31.2–38.5)	35.9 (32.2–39.8)	31.5 (26.2–36.6)	
	Intermediate	37.8 (34.9–40.8)	38.8 (35.6–42.0)	36.8 (33.0–40.5)	39.5 (35.6–43.3)	36.1 (30.5–41.7)	
	High	41.9 (38.9–45.2)	40.9 (37.7–44.1)	38.4 (34.6–42.0)	38.3 (34.2–42.6)	40.2 (35.0–45.0)	
3	Low	34.4 (31.4–37.5)	31.8 (28.8–34.7)	31.6 (28.4–35.5)	30.5 (27.1–34.2)	27.4 (22.9–32.3)	
	Intermediate	28.7 (25.9–31.3)	28.8 (25.7–31.8)	24.3 (21.2–27.5)	24.8 (21.5–28.4)	22.8 (18.3–27.8)	
	High	22.8 (20.1–25.6)	24.5 (21.8–27.1)	21.5 (18.6–24.5)	22.4 (19.3–25.8)	19.0 (15.2–22.9)	
4 *highest risk*	Low	17.9 (15.9–20.1)	18.3 (16.0–20.7)	16.0 (13.3–18.8)	16.8 (13.8–19.6)	19.4 (14.7–23.8)	
	Intermediate	13.2 (11.1–15.1)	13.3 (11.1–15.6)	13.4 (11.0–15.7)	14.8 (12.3–17.5)	10.2 (7.2–13.5)	
	High	12.5 (10.5–14.4)	7.5 (5.9–9.2)	9.4 (7.3–11.6)	12.6 (10.2–15.2)	11.4 (8.2–14.6)	

^a^The risk factor accumulation score was formed from the following four risk factors: current smoking, obesity, elevated total cholesterol and hypertension. The classes were as follows: (1) no risk factors, (2) one risk factor, (3) two risk factors, and (4) three or four risk factors.

^b^Interaction between education and study year.

**Table 5:** Risk factor accumulation score^a^ by marital status in FINRISK (FR) 1997–2012 and FinHealth (FH) 2017 surveys. The age range of included participants was 25–64 years.

Risk factor score^a^	Marital status^b^	FR97	FR02	FR07	FR12	FH2017	p for interaction^c^
**Men** (n = 11616)							0.06
1 *lowest risk*	Married	11.4 (10.0–12.8)	11.2 (9.6–12.7)	14.1 (12.2–16.0)	15.2 (13.4–17.1)	16.2 (13.3–19.4)	
	Single	12.9 (10.5–15.2)	13.4 (10.7–16.3)	12.8 (10.0–15.9)	13.7 (10.5–17.4)	17.8 (13.2–22.8)	
2	Married	30.2 (28.3–32.1)	33.7 (31.7–35.8)	34.5 (31.8–37.1)	31.5 (29.1–34.0)	35.9 (32.6–40.1)	
	Single	27.1 (23.4–30.9)	26.8 (22.8–30.8)	24.4 (20.2–28.7)	32.4 (27.9–37.1)	32.7 (26.1–39.8)	
3	Married	38.0 (35.9–40.1)	34.8 (32.6–37.1)	32.1 (29.7–34.6)	33.7 (31.1–36.4)	30.7 (26.8–34.3)	
	Single	34.0 (30.4–37.9)	30.2 (26.3–34.3)	39.0 (34.4–43.5)	31.6 (27.0–36.5)	27.0 (20.4–33.0)	
4 *highest risk*	Married	20.4 (18.8–21.9)	20.3 (18.5–21.9)	19.3 (17.4–21.3)	19.6 (17.6–21.7)	17.2 (13.6–20.8)	
	Single	26.0 (22.8–29.1)	29.6 (25.7–33.5)	23.8 (19.8–27.7)	22.2 (18.2–26.0)	22.6 (16.9–29.0)	
**Women** (n = 13380)							0.53
1 *lowest risk*	Married	19.2 (17.6–20.8)	22.0 (20.4–23.7)	26.5 (24.3–28.7)	22.7 (20.7–24.8)	29.5 (26.3–32.7)	
	Single	16.5 (13.8–19.4)	17.3 (14.3–20.1)	19.8 (16.5–23.3)	19.5 (16.2–22.9)	21.9 (17.2–26.8)	
2	Married	38.5 (36.6–40.3)	37.4 (35.4–39.6)	36.8 (34.4–39.3)	38.2 (35.8–40.8)	34.3 (30.8–37.8)	
	Single	37.0 (33.4–40.6)	39.2 (35.5–43.0)	36.2 (31.9–40.2)	36.6 (32.6–40.8)	41.5 (34.9–47.1)	
3	Married	28.6 (26.8–30.4)	27.7 (25.9–29.4)	24.8 (22.7–26.8)	25.4 (23.2–27.6)	23.9 (21.1–27.1)	
	Single	29.4 (26.1–32.7)	29.7 (26.4–33.1)	28.1 (24.3–32.3)	27.1 (23.2–30.6)	19.7 (15.0–25.0)	
4 *highest risk*	Married	13.7 (12.3–15.0)	12.9 (11.5–14.3)	11.9 (10.4–13.4)	13.7 (12.1–15.4)	12.3 (10.2–14.6)	
	Single	17.1 (14.7–19.7)	13.8 (11.3–16.4)	15.9 (13.4–18.9)	16.8 (13.8–20.2)	16.9 (12.8–21.9)	

^a^The risk factor accumulation score was formed from the following four risk factors: current smoking, obesity, elevated total cholesterol and hypertension. The classes were as follows: (1) no risk factors, (2) one risk factor, (3) two risk factors, and 4) three or four risk factors.

^b^Marital status classes were as follows: married = living with a spouse/partner (i.e. married, cohabiting), and single = living without a spouse (i.e. single, separated or divorced, widowed).

^c^Interaction between marital status and study year.

The statistical analyses were performed using R^19^. R-package ‘survey’ was used for analysing complex survey samples and R-package ‘nnet’ was used for running MLR models^20,21^.

## Results

### Risk factor combinations

The proportion of those with no elevated risk factors increased from 12.6 to 17.9% among men (p = 0.001) and from 19.3 to 25.8% among women from 1997 to 2017 (p < 0.001) (Tables 2 and 3). Among men, 2.6–3.9% of the study participants had elevated levels for all four risk factors over the years 1997–2017, whereas among women, the range was 1.2–2.0%. There were significant changes in several risk factor combinations. The prevalences for all 16 combinations of the four selected risk factors (current smoking, obesity, elevated cholesterol and hypertension) for 1997–2017 are shown in Tables 2 and 3 for men and women, respectively.

### Risk factor accumulation score by sex, education and marital status

Overall, the four-category risk factor accumulation score was more favourable among women than among men, as the lowest risk category was more common among women and the highest risk category among men (Fig. 1, Tables 4 and 5).

The risk factor accumulation score was most favourable among participants in the highest education tertile in both sexes (Fig. 1, Table 4). The change in educational differences in risk factor accumulation score during the study period was not significant. The lowest risk factor accumulation score class (i.e. participants with no risk factors) became more common from 1997 to 2017, and the change was significant in low and intermediate education groups among women but only in the intermediate education group of men. It seemed that the intermediate education group moved closer to the highest education group between 1997 and 2017, especially among men. This was true both in the lowest and the highest risk factor accumulation score groups.

The risk factor accumulation score tended to be slightly more favourable among participants living with a spouse or partner compared to those living without a spouse. The difference was significant for men in the highest risk category in 1997 and 2002 and for women in 2007, and for women in the lowest risk category in 2002 and 2007 (Fig. 1, Table 5). The change in the differences by marital status in the risk factor accumulation score was borderline significant among men (p = 0.06) but not among women (p = 0.53). In 2007, the risk factor accumulation score among men living without a spouse deviated somewhat from the other studies with higher prevalence in the third risk factor accumulation category, which may explain the reasonably low p value.

## Discussion

The repeated cross-sectional studies between 1997 and 2017 from Finland indicate an overall transition towards a more favourable risk factor situation, when smoking, obesity, elevated total cholesterol and hypertension are considered. The prevalence of those with none of the four selected risk factors increased in both sexes, and the proportion of those with all four risk factors remained low. Overall, the risk factor accumulation score, which combined the four risk factors, was more favourable among women than men. Also higher education was associated with a more favourable risk factor accumulation score throughout the study years. However, during the study period, the risk factor accumulation score of the intermediate education group approached the highest education group, especially among men. Thereby, the risk factors tended to accumulate especially in the lowest education tertile towards the end of the study period.

The tendency of unhealthy behaviours and biological risk factors to accumulate in lower education groups is in line with numerous studies from several countries^22–26^. The direction of the relationship is, however, not unequivocal. Social gradient, social exclusion and unemployment are interrelated and are all included among the major social determinants of health^27^. On the other hand, poor health may limit the ability to find and retain paid work, and the stigma associated with ill-health may restrict social participation. Thus, poor health may also contribute to exclusionary processes^28^.

We chose education as a sociodemographic indicator for both theoretical and practical reasons. Education has traditionally been considered as a functional sociodemographic indicator as education is likely to reflect a persons’ cognitive skills and possession of knowledge, which may make them more receptive to health messages, more competent in processing health-related information and more motivated to behavior change^29,30^. At the same time, it helps determine a person’s occupational level and income, and therefore shares some of the health effects of these indicators^29^. Education usually precedes occupation and income, and it can also be assumed a more stable indicator than occupation or income in our data of adults at least 25 years of age. Furthermore, questions on education are often more accurately and more completely responded in surveys compared to the more sensitive questions on income^31–33^. Regarding the studies included in our analyses, education was also one of the most comparably measured sociodemographic indicators across the studies. However, as our data included subjects born between 1948 and 1992, we used relative education where the years of education were divided into tertiles based on birth years. Yet, it may be that for example the assumed relationship between education and occupational level may have changed during the study period as a university degree no longer guarantees a job in good position as it did some decades ago in Finland.

The risk factor accumulation score was slightly more favourable among those living with a spouse compared to those living without a spouse. Significant differences by marital status were observed in the highest risk factor accumulation score (i.e. persons with three or four risk factors), among both sexes and in the lowest risk factor accumulation score (no risk factors) among women. In 1997 and 2002 among men and in 2007 among women, the prevalence of the highest risk factor accumulation score category was significantly higher among those living without a spouse than among those living with a spouse. The difference attenuated somewhat along the later study years. Our results are in line with earlier studies in which being single or unmarried has been found to be associated with the accumulation of risk factors or unhealthy behaviours^23,24^, and furthermore with increased odds of CVD, coronary heart disease (CHD), and CHD and stroke mortality^34^. Examples of mechanisms that have been suggested to account for the protective effect of marital status include spousal support leading to earlier recognition of warning symptoms as well as encouragement to healthy lifestyle choices and better adherence to treatment^34^.

There are some methodological limitations in regards to our study. We acknowledge that the four-class risk factor accumulation score which we used simplifies the complexity of issues related to each of the included risk factors. First of all, the dichotomization of the lifestyle factors and biological risk factors includes problems as persons close to both sides of the cut-off may actually be quite similar. However, we used established cut-offs for BMI, elevated total cholesterol and blood pressure.

Furthermore, we included the use of antihypertensive or cholesterol-lowering medicines in the definitions of the respective risk factors. It might be discussed, if only the blood pressure or cholesterol levels without information on medication use should have been considered, as decreasing the level of blood pressure or cholesterol also decreases the risk of diseases or events. However, although the use of antihypertensive or cholesterol-lowering medicines lowers the risk of clinical outcomes, persons on antihypertensive or cholesterol-lowering medication are still considered as suffering from hypertension or hyperlipidemia. Furthermore, we inferred that the use of these medicines may indicate an underlying factor that may disclose the person’s elevated risk status, even though the actual blood pressure or cholesterol levels would have been in the normal range at the time of measurement. The cholesterol or blood pressure levels may have been elevated for a longer time period before starting the medication, and thereby reducing the actual risk factor levels may not fully eliminate the long-term effects already caused by the elevated levels. The reason for the use of lipid lowering and antihypertensive medications may also be primary prevention due to some other health condition or disease event. However, we performed the analyses also without including the use of medicines in the definition. These results are included in Supplementary Tables 1–4. The risk factor score as a whole shifted slightly to a more favourable direction. However, excluding the use of antihypertensive or cholesterol-lowering medicines from the definition did not markedly affect the interpretation of the results. During this process, we also examined the subgroup of subjects on medication but with elevated blood pressure or cholesterol levels more thoroughly and included these analyses as Supplementary Table 5. The proportion of subjects with elevated blood pressure and using antihypertensive medicines was close to 8% and we could also present the proportions of this subgroup by sex and education (Supplementary Tables 5 and 6).The proportion of subjects with elevated total cholesterol and using cholesterol-lowering medicines was only close to 2% and thereby, more elaborate analyses were not feasible for this group (Supplementary Table 5).

We also performed latent class analyses (LCA) with R packages poLCA and BayesLCA to identify subtypes of the combinations of the four risk factors. The analyses resulted in remarkably uneven latent classes with a great number of participants in a class with a low risk factor profile, probably because the risk factors were so evenly distributed. Therefore, we excluded the analyses from this study.

In addition to differences in health behaviours and biological risk factors across sociodemographic groups, social inequality in the ability to make a behavior change also exists. In a recent study in Denmark, considerable differences between social groups in the number of barriers to lifestyle change were observed^35^. To reduce sociodemographic differences in risk factor occurrence, more targeted measures of prevention which would be effective especially in lower socioeconomic groups are needed. This need for tailored initiatives is strongly supported by previous studies^35,36^.

Overall, a favourable trend of diminishing risk factor prevalence was seen. The tendency of accumulation of major CVD risk factors among the least educated subjects remained from 1997 to 2017.

## Supplementary information

Supplementary information.

## Data Availability

The datasets analysed during the current study are not publicly available due to restrictions based in the General Data Protection Regulation (GDPR) on sensitive data such as personal health data. The access to the data may be requested through the Finnish Institute for Health and Welfare (THL) Biobank (https://thl.fi/en/web/thl-biobank/for-researchers).
