# Supplementary material for: Inequalities by education and marital status in the co-occurrence of cardiovascular risk factors in Finland persisted between 1997–2017
Source: Sci Rep. 2020 Jun 4;10:9123. doi: 10.1038/s41598-020-65959-1 (PMC7272447; doi:10.1038/s41598-020-65959-1)
Supplement: Supplementary file 1 — Supplementary information. [file 41598_2020_65959_MOESM1_ESM.pdf]

Supplementary Tables 1, 2, 3, 4, 5 and 6

for *"Inequalities by education and marital status in the co-occurrence of cardiovascular risk factors in Finland persisted between 1997–2017"*

in Scientific Reports

Paalanen L[1], Härkänen T[1], Kontto J[1], Tolonen H[1]

[1]Department of Public Health Solutions, Finnish Institute for Health and Welfare (THL), P.O. Box 30, FI-00271 Helsinki, Finland

E-mail address of the corresponding author:

laura.paalanen@thl.fi

Supplementary table 1

Prevalence of risk factor combinations (%) among 25–64-year-old men in FINRISK (FR) 1997–2012 and FinHealth (FH) 2017 surveys (n=11,633)

Number of risk factors	Current smoking	Obesity[a]	Elevated cholesterol[b]	Elevated blood pressure[c]		FR97	FR02	FR07	FR12	FH2017	p
4	+	+	+	+		2.6	3.5	1.8	2.7	2.1	0.01
					Total	2.6	3.5	1.8	2.7	2.1	
3	+	+	+	-		1.7	1.8	2.0	1.9	1.4	0.88
	+	+	-	+		0.6	0.8	1.0	1.1	1.6	0.19
	+	-	+	+		9.0	7.7	6.8	4.8	3.5	<0.001
	-	+	+	+		6.8	6.1	5.3	5.6	3.9	0.02
					Total	18.1	16.4	15.1	13.4	10.4	
2	+	+	-	-		0.4	0.9	1.1	1.0	1.6	0.06
	+	-	+	-		10.8	12.2	8.6	8.4	4.5	<0.001
	+	-	-	+		2.7	2.2	2.7	2.9	1.7	0.29
	-	+	+	-		2.6	3.4	2.9	2.5	6.2	<0.001
	-	+	-	+		1.7	2.1	3.3	2.7	3.0	0.01
	-	-	+	+		18.1	12.0	13.5	14.0	11.8	<0.001
					Total	36.3	32.8	32.1	31.5	28.8	
1	+	-	-	-		5.5	7.8	7.9	6.1	7.0	0.03
	-	+	-	-		0.9	2.2	1.4	3.0	3.6	<0.001
	-	-	+	-		17.6	18.6	17.9	19.1	22.2	0.10
	-	-	-	+		5.6	5.2	7.7	5.8	5.7	0.05
					Total	29.6	33.8	34.9	34.0	38.5	
0	-	-	-	-		13.2	13.7	16.2	18.6	20.1	<0.001
					Total	13.2	13.7	16.2	18.6	20.1	

[a]BMI≥30 kg/m^2

[b]Serum total cholesterol ≥5.0 mmol/l **(use of cholesterol-lowering medicines not considered)**

[c]Systolic blood pressure ≥140 mmHg or diastolic blood pressure ≥90 mmHg **(use of antihypertensive medicines not considered)**

Supplementary table 2

Prevalence of risk factor combinations (%) among 25–64-year-old women in FINRISK (FR) 1997–2012 and FinHealth (FH) 2017 surveys (n=13,403)

Number of risk factors	Current smoking	Obesity[a]	Elevated cholesterol[b]	Elevated blood pressure[c]		FR97	FR02	FR07	FR12	FH2017	p
4	+	+	+	+		1.4	1.0	1.2	1.3	1.3	0.75
					Total	1.4	1.0	1.2	1.3	1.3	
3	+	+	+	-		1.4	1.8	1.6	1.5	2.3	0.58
	+	+	-	+		0.1	0.4	0.9	0.4	0.1	0.17
	+	-	+	+		3.5	2.4	1.9	2.4	1.9	0.03
	-	+	+	+		7.2	5.3	5.2	5.6	3.6	<0.001
					Total	12.2	9.9	9.6	9.9	7.9	
2	+	+	-	-		0.7	1.1	1.0	0.9	1.2	0.69
	+	-	+	-		8.8	7.7	6.6	6.4	5.0	0.001
	+	-	-	+		0.9	1.2	1.2	0.6	1.0	0.51
	-	+	+	-		4.0	4.5	4.5	5.4	7.3	<0.01
	-	+	-	+		1.3	1.3	1.7	1.6	1.8	0.67
	-	-	+	+		12.6	9.9	8.5	9.6	7.1	<0.001
					Total	28.3	25.7	23.5	24.5	23.4	
1	+	-	-	-		7.0	8.9	8.5	7.4	6.5	0.09
	-	+	-	-		1.9	2.2	2.8	3.3	4.6	<0.001
	-	-	+	-		26.4	24.6	23.0	26.2	26.5	0.06
	-	-	-	+		3.4	5.0	5.2	3.8	2.5	0.001
					Total	38.7	40.7	39.5	40.7	40.1	
0	-	-	-	-		19.3	22.5	26.1	23.8	27.3	<0.001
					Total	19.3	22.5	26.1	23.8	27.3	

[a]BMI≥30 kg/m^2

[b]Serum total cholesterol ≥5.0 mmol/l **(use of cholesterol-lowering medicines not considered)**

[c]Systolic blood pressure ≥140 mmHg or diastolic blood pressure ≥90 mmHg **(use of antihypertensive medicines not considered)**

Supplementary table 3

Risk factor accumulation score[a] by education in FINRISK (FR) 1997–2012 and FinHealth (FH) 2017 surveys. The age range of included participants was 25–64 years.

Risk factor score[a]	Education level	FR97	FR02	FR07	FR12	FH2017	p for interaction[b]
**Men** (n=11,540)							0.89
1	Low	9.5 (7.5-11.7)	8.7 (6.7-10.8)	9.7 (7.0-12.6)	13.1 (10.0-16.1)	13.8 (9.3-18.4)	
lowest risk	Intermediate	11.6 (9.3-14.0)	11.1 (8.7-13.7)	16.1 (13.0-19.2)	18.1 (14.7-21.8)	22.4 (17.9-27.3)	
	High	16.1 (13.9-18.4)	19.5 (16.8-22.5)	20.1 (17.1-23.4)	20.3 (16.8-24.0)	19.9 (15.5-24.7)	
2	Low	26.8 (23.8-29.7)	29.9 (26.7-33.2)	32.2 (28.1-36.5)	31.3 (27.8-35.2)	31.8 (25.8-38.4)	
	Intermediate	28.9 (25.9-32.1)	35.2 (31.7-39.2)	32.6 (28.7-36.3)	34.2 (29.8-38.3)	35.7 (30.4-42.1)	
	High	34.3 (31.2-37.5)	35.6 (32.3-38.9)	39.3 (35.3-43.3)	39.7 (34.9-44.5)	45.9 (40.2-51.6)	
3	Low	38.9 (35.7-42.2)	36.5 (33.0-40.0)	36.5 (32.3-40.7)	35.2 (31.2-39.6)	33.2 (26.7-39.8)	
	Intermediate	37.7 (34.6-40.7)	33.6 (30.2-37.1)	33.4 (29.7-37.1)	31.7 (27.8-35.9)	29.5 (24.3-34.6)	
	High	33.1 (29.9-35.9)	28.7 (25.5-32.3)	28.8 (25.4-32.5)	28.0 (24.0-32.2)	24.2 (19.4-29.1)	
4	Low	24.8 (22.1-27.7)	24.8 (21.7-28.0)	21.5 (18.1-25.3)	20.4 (17.3-23.5)	21.2 (14.4-27.9)	
highest risk	Intermediate	21.8 (19.2-24.4)	20.0 (17.3-22.6)	17.9 (15.0-20.7)	16.0 (12.9-19.2)	12.4 (9.0-16.3)	
	High	16.5 (14.2-19.0)	16.2 (13.4-18.7)	11.8 (9.2-14.4)	12.0 (9.4-15.0)	10.0 (6.7-13.9)	
**Women** (n=13,277)							0.09
1	Low	12.6 (10.5-15.1)	16.2 (13.8-18.8)	18.7 (15.7-21.9)	18.2 (15.0-21.5)	22.7 (18.1-27.7)	
lowest risk	Intermediate	20.4 (18.2-22.6)	19.8 (17.2-22.4)	27.6 (24.4-31.0)	21.5 (18.0-25.1)	31.9 (26.8-37.3)	
	High	22.9 (20.4-25.6)	28.3 (25.5-31.1)	32.3 (29.0-35.7)	27.8 (24.4-31.1)	32.6 (28.0-37.5)	
2	Low	36.9 (33.7-39.9)	36.1 (33.0-39.3)	36.6 (32.8-40.2)	39.3 (35.3-43.4)	33.5 (28.2-38.9)	
	Intermediate	39.1 (36.2-42.2)	41.1 (37.8-44.3)	38.6 (34.8-42.5)	42.1 (38.0-46.1)	39.5 (34.1-45.4)	
	High	42.6 (39.5-45.9)	42.4 (39.2-45.7)	40.6 (36.7-44.4)	39.7 (35.7-44.0)	41.4 (36.2-46.0)	
3	Low	34.2 (31.2-37.4)	31.4 (28.5-34.3)	31.8 (28.6-35.8)	29.1 (25.3-32.8)	29.9 (25.2-35.3)	
	Intermediate	28.5 (25.6-31.1)	27.8 (24.7-30.8)	22.0 (18.9-25.2)	24.9 (21.7-28.3)	21.4 (17.1-26.2)	
	High	22.6 (19.9-25.3)	23.0 (20.4-26.0)	19.4 (16.5-22.8)	22.5 (19.1-25.7)	17.5 (13.8-21.6)	
4	Low	16.2 (14.2-18.4)	16.3 (14.0-18.5)	12.8 (10.4-15.5)	13.4 (10.7-16.1)	13.9 (9.9-18.0)	
highest risk	Intermediate	12.0 (10.1-13.9)	11.4 (9.2-13.6)	11.7 (9.6-13.9)	11.5 (9.1-13.9)	7.2 (4.5-10.1)	
	High	11.9 (9.8-13.7)	6.3 (4.9-7.9)	7.8 (5.9-9.8)	10.0 (7.7-12.5)	8.4 (5.5-11.5)	

[a]The risk factor accumulation score was formed from the following four risk factors: current smoking, obesity, elevated total cholesterol and elevated blood pressure **(based on measured cholesterol and blood pressure values only; use of cholesterol-lowering or antihypertensive medicines not considered)**. The classes were as follows: 1) no risk factors, 2) one risk factor, 3) two risk factors, and 4) three or four risk factors.

[b]Interaction between education and study year

Supplementary table 4

Risk factor accumulation score[a] by marital status in FINRISK (FR) 1997–2012 and FinHealth (FH) 2017 surveys. The age range of included participants was 25–64 years.

Risk factor score[a]	Marital status[b]	FR97	FR02	FR07	FR12	FH2017	p for interaction[c]
**Men** (n=11,616)							0.08
1	Married	12.2 (10.8-13.6)	12.6 (11.0-14.1)	15.8 (13.9-17.7)	18.0 (16.1-20.2)	18.3 (15.2-21.6)	
lowest risk	Single	13.4 (10.9-15.8)	14.4 (11.6-17.5)	15.0 (11.9-18.4)	15.6 (12.1-19.6)	20.0 (15.1-25.7)	
2	Married	30.9 (28.9-32.9)	35.1 (33.0-37.4)	37.4 (34.8-39.9)	34.3 (31.7-36.8)	38.9 (35.4-42.9)	
	Single	28.1 (24.5-31.8)	28.7 (24.5-32.8)	27.9 (23.7-32.5)	36.2 (31.5-41.0)	35.9 (28.9-43.3)	
3	Married	37.5 (35.5-39.6)	34.2 (32.0-36.5)	30.9 (28.5-33.3)	32.1 (29.5-34.6)	29.4 (25.7-33.1)	
	Single	33.1 (29.3-36.9)	29.4 (25.6-33.5)	37.6 (33.1-41.8)	30.9 (25.9-35.5)	27.5 (21.6-33.4)	
4	Married	19.4 (17.8-20.9)	18.1 (16.4-19.7)	16.0 (14.1-17.8)	15.6 (13.9-17.5)	13.4 (10.0-17.0)	
highest risk	Single	25.4 (22.1-28.5)	27.5 (23.6-31.2)	19.5 (15.8-23.4)	17.4 (13.5-21.3)	16.6 (11.5-22.5)	
**Women** (n=13,380)							
							0.70
1	Married	19.3 (17.8-20.9)	22.8 (21.2-24.5)	28.1 (25.9-30.3)	23.6 (21.6-25.8)	31.2 (27.9-34.5)	
lowest risk	Single	16.5 (13.8-19.3)	18.1 (15.0-20.9)	21.4 (18.0-25.0)	20.8 (17.3-24.1)	24.0 (18.7-29.1)	
2	Married	39.8 (37.8-41.7)	39.4 (37.4-41.6)	38.5 (35.8-40.9)	40.7 (38.2-43.2)	36.6 (33.0-40.1)	
	Single	38.1 (34.5-41.8)	40.6 (36.8-44.4)	38.8 (34.3-42.9)	39.1 (34.4-43.4)	43.8 (37.5-49.6)	
3	Married	28.3 (26.5-30.1)	26.6 (24.8-28.3)	23.6 (21.6-25.7)	25.0 (22.8-27.1)	23.0 (20.1-26.0)	
	Single	29.5 (26.4-32.9)	29.2 (25.8-32.7)	26.4 (22.6-30.3)	26.5 (22.8-30.4)	21.2 (16.4-26.5)	
4	Married	12.5 (11.2-13.9)	11.1 (9.8-12.5)	9.8 (8.4-11.2)	10.7 (9.2-12.3)	9.2 (7.3-11.2)	
highest risk	Single	15.9 (13.5-18.5)	12.1 (9.8-14.5)	13.5 (10.9-16.3)	13.6 (10.9-16.8)	11.0 (7.2-15.6)	

[a]The risk factor accumulation score was formed from the following four risk factors: current smoking, obesity, elevated total cholesterol and elevated blood pressure **(based on measured cholesterol and blood pressure values only; use of cholesterol-lowering or antihypertensive medicines not considered)**. The classes were as follows: 1) no risk factors, 2) one risk factor, 3) two risk factors, and 4) three or four risk factors.

[b]Marital status classes were as follows: married = living with a spouse/partner (i.e. married, cohabiting), and single = living without a spouse (i.e. single, separated or divorced, widowed).

[c]Interaction between marital status and study year

Supplementary table 5

Crosstabulation of subjects with elevated total cholesterol[a] vs. using cholesterol-lowering medication and subjects with elevated blood pressure[b] vs. using antihypertensive medicines in FINRISK (FR) 1997–2012 and FinHealth (FH) 2017 surveys[c]. The age range of included participants was 25–64 years (11,616 men and 13,380 women).

	FR97	FR02	FR07	FR12	FH2017
**Total cholesterol**					
No elevated level & no medication	2139 (30.3%)	2214 (34.7%)	1852 (38.8%)	1487 (34.0%)	927 (37.6%)
No elevated level & on medication	42 (0.6%)	171 (2.7%)	237 (4.9%)	242 (5.5%)	159 (6.4%)
Elevated level & no medication	4722 (67.0%)	3819 (59.9%)	2512 (52.6%)	2497 (57.1%)	1307 (53.0%)
Elevated level & on medication	145 (2.1%)	171 (2.7%)	173 (3.6%)	147 (3.4%)	73 (3.0%)
**Blood pressure**					
No elevated level & no medication	4061 (57.6%)	3872 (60.7%)	2826 (59.2%)	2612 (59.7%)	1536 (62.3%)
No elevated level & on medication	185 (2.6%)	244 (3.8%)	248 (5.2%)	275 (6.3%)	179 (7.3%)
Elevated level & no medication	2222 (31.5%)	1751 (27.5%)	1301 (27.3%)	1128 (25.8%)	548 (22.2%)
Elevated level & on medication	580 (8.2%)	508 (8.0%)	399 (8.4%)	358 (8.2%)	203 (8.2%)

[a]Serum total cholesterol ≥5.0 mmol/l

[b]Systolic blood pressure ≥140 mmHg or diastolic blood pressure ≥90 mmHg

[c]Crude rates

Supplementary table 6

The proportion of subjects with elevated blood pressure[a] and using antihypertensive medicines by sex and by education in FINRISK (FR) 1997–2012 and FinHealth (FH) 2017 surveys[b]. The age range of included participants was 25–64 years (11,616 men and 13,380 women).

	FR97	FR02	FR07	FR12	FH2017
**Blood pressure**					
**Sex**					
Men	9.5%	8.7%	9.4%	9.5%	10.2%
Women	7.1%	7.3%	7.5%	7.1%	6.5%
**Education**					
Low	35.4%	36.5%	37.0%	30.6%	37.3%
Intermediate	29.7%	31.5%	34.3%	38.5%	33.8%
High	34.9%	31.9%	28.7%	30.9%	28.9%

[a]Systolic blood pressure ≥140 mmHg or diastolic blood pressure ≥90 mmHg

[b]Crude rates
